# The association between a body shape index and elevated urinary albumin–creatinine ratio in Chinese community adults

**DOI:** 10.3389/fendo.2022.955241

**Published:** 2022-07-28

**Authors:** Yue Zhang, Wenxing Gao, Binqi Li, Yang Liu, Kang Chen, Anping Wang, Xulei Tang, Li Yan, Zuojie Luo, Guijun Qin, Lulu Chen, Qin Wan, Zhengnan Gao, Weiqing Wang, Guang Ning, Yiming Mu

**Affiliations:** ^1^ Medical School of Chinese People's Liberation Army, Beijing, China; ^2^ Department of Endocrinology, The First Clinical Medical Center of Chinese People’s Liberation Army General Hospital, Beijing, China; ^3^ School of Medicine, Nankai University, Tianjin, China; ^4^ Department of Endocrinology, The First Hospital of Lanzhou University, Lanzhou, China; ^5^ Sun Yat-sen Memorial Hospital, Sun Yat-sen University, Guangzhou, China; ^6^ Department of Endocrinology, The First Affiliated Hospital of Guangxi Medical University, Nanning, China; ^7^ Department of Endocrinology, The First Affiliated Hospital of Zhengzhou University, Zhengzhou, China; ^8^ Union Hospital, Tongji Medical College, Wuhan, China; ^9^ Department of Endocrinology, Affiliated Hospital of Luzhou Medical College, Luzhou, China; ^10^ Department of Endocrinology, Dalian Municipal Central Hospital, Dalian, China; ^11^ Ruijin Hospital, Shanghai Jiao Tong University School of Medicine, Shanghai, China

**Keywords:** a body shape index, albuminuria, visceral obesity, chronic kidney disease, body mass index

## Abstract

**Background:**

Obesity, especially visceral obesity, seems to be one of the most decisive risk factors for chronic kidney disease. A Body Shape Index (ABSI) is an emerging body size measurement marker of visceral obesity. This study aimed to explore whether ABSI is associated with albuminuria in Chinese community adults.

**Methods:**

This cross-sectional study enrolled 40,726 participants aged 40 or older from seven provinces across China through a cluster random sampling method. ABSI was calculated by body mass index, waist circumference, and height. Increased albuminuria was defined as urinary albumin–creatinine ratio (UACR) ≥ 30 mg/g, indicating kidney injury. For ABSI, we divided it by quartile cutoff points and tried to determine the association between ABSI levels and UACR by multiple regression analysis. DAG (Directed Acyclic Graph) was plotted using literature and expert consensus to identify potential confounding factors.

**Results:**

The average age of subjects with elevated UACR was 61.43 ± 10.07, and 26% were men. The average age of subjects with normal UACR was 57.70 ± 9.02, and 30.5% were men. Multiple logistic regression analysis was conducted and demonstrated that the ABSI quartiles were related to elevated UACR positively (OR [95% CI] Q2 vs. Q1: 1.094 [1.004, 1.197]; OR [95% CI] Q3 vs. Q1: 1.126 [1.030, 1.231]; OR [95% CI] Q4 vs. Q1: 1.183 [1.080, 1.295], *p* for trend < 0.001) after adjustments for confounding factors. The stratified analysis further showed that with the mounting for ABSI levels, elevated UACR more easily occurred in the people characterized by the elderly, men, and hypertension.

**Conclusions:**

In Chinese community adults, people with higher ABSI levels can be deemed as high-risk individuals with UACR elevation, and it will be beneficial for them to lose weight and significantly reduce visceral fat.

## Introduction

Nowadays, chronic kidney disease (CKD) has been changed into a global public health threat. The estimated prevalence of CKD was 9.1% worldwide in 2017, ranking as the 12th leading cause of death (1). The onset of CKD is insidious, and it was easily advanced to end-stage renal disease (ESRD), which has a poor prognosis and high mortality, posing a heavy burden on public health and the economy (2). UACR, as a sensitive marker of early kidney injury, is currently used in clinical screening for CKD to identify high-risk populations (3).

There is a remarkable phenomenon that the prevalence of obesity in patients with CKD is high, mounting from 38.1% in 1999–2002 to 44.1% in 2011–2014 in the United States (4). A large population of European survey demonstrated that among the risk factors of new CKD, obesity is one of the strongest one (5). Body mass index (BMI), used for obesity measurement most commonly (6), remains limited by its inability to provide information on fat distribution and distinguish fat accumulation from muscle (7). Krakauer invented ABSI (8), which consists of waist circumference (WC), height, and BMI. High ABSI values correspond to high visceral fat, which not only predicts the risk of premature death independent of BMI, but also is a marker of abdominal obesity and insulin resistance in men (9).

pt?>A cohort study of 5,438 urban residents in Japan found that ABSI can predict subjects at risk of renal function decline more effectively than WC (10). A cross-sectional survey of 7,053 older people in South Korea showed that the ABSI had a better capacity to discriminate the CKD stage than BMI (11). However, to our knowledge, evidence of the relationship between ABSI and UACR in the large-sample population is lacking. Therefore, in this study, we collected data from 40,726 Chinese adults to explore the relationship between ABSI and albuminuria and identify high-risk individuals as early as possible to provide evidence for CKD prevention.

## Methods

### Participants and study design

**Figure 1:**
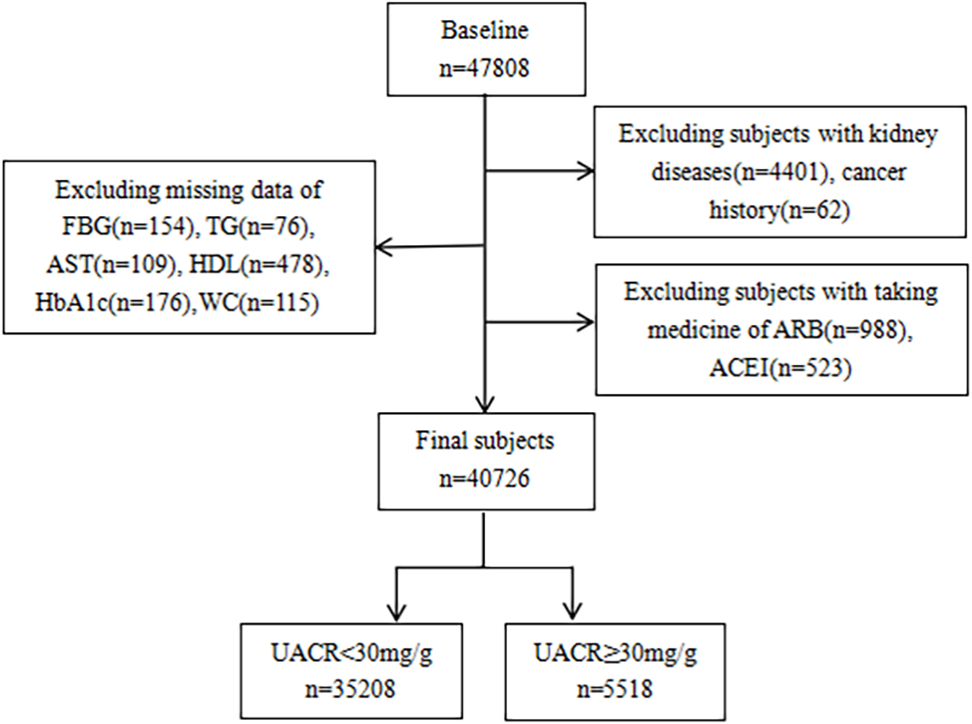
Flowchart of the study population.

Data from the cross-sectional study came from the REACTION (China’s Risk Evaluation of cAncers in Chinese diabeTic Individuals, a lONgitudinal study), which recruited 47,808 individuals over the age of 40 from May to December 2011 in seven geographically diverse regional centers in China (Zhengzhou, Dalian, Luzhou, Shanghai, Wuhan, Guangzhou, and Lanzhou) (12). Participants with a diagnosis of primary kidney disease, a history of malignancy, prior use of antihypertensive medications (angiotensin-converting enzyme inhibitors or angiotensin receptor blockers), or lack of significant data were excluded. Finally, we enrolled 33,303 participants (**Figure 1**). The Clinical Research Ethics Committee approved this study of Ruijin Hospital, affiliated with Shanghai Jiao Tong University School of Medicine (2014-25). This study was performed according to the Declaration of Helsinki. All participants, before participation, provided informed consent.

### Data collection

Trained investigators collected basic information about participants through standardized questionnaires, including age, gender, history of underlying diseases, medication history, lifestyle, smoking habits, and drinking habits. Anthropometric measurements include weight, height, WC, diastolic blood pressure (DBP), and systolic blood pressure (SBP). Participants removed their clothes and shoes before the measurement. Blood pressure was measured three times with a mercury sphygmomanometer and averaged. All subjects had to sit still for at least 5 min before the measurement. The definition of WC is the abdominal circumference connecting the lower margin of the thorax to the midpoint of the iliac crest, the hip circumference (HC) was defined as the length of the hip joint protrusion horizontally. Fasting blood samples and morning urine were collected after 10 h of fasting. Biochemical parameters included aspartate transferase (AST), alanine transferase (ALT), glutamyltransferase (GGT), fasting blood glucose (FBG), 2 h postprandial blood glucose (PBG), rapid insulin determination (0 min, 120 min), glycosylated hemoglobin A1c (HbA1c), triglyceride (TG), total cholesterol (TC), serum creatinine (Scr), high-density lipoprotein cholesterol (HDL-C), and low-density lipoprotein cholesterol (LDL-C).

### Definition of variables

BMI is the weight (kg) divided by height squared (m^2^). ABSI was calculated by WC (m)/[BMI^2/3^ (kg/m^2^) × height^1/2^ (m)]. Waist-to-hip ratio (WHR) and waist-to-height ratio (WHtR) are WC divided by HC and WC divided by height, respectively. Smoking habits were defined as non-smoking, occasional smoking (less than one cigarette per day or less than seven cigarettes per week), and regular smoking (at least one cigarette per day). Normal blood pressure is defined as SBP of less than 120 and DBP of less than 80; hypertension was defined as SBP greater than 140 or DBP greater than 90; between the two categories is pre-hypertension. UACR was calculated as urinary albumin (mg)/urinary creatinine (g). According to the KDIGOCKD guidelines (13), UACR ≥ 30 mg/g was the definition of increased proteinuria, suggesting kidney damage. The UACR group was divided into two groups: normal proteinuria group: UACR < 30 mg/g and increased proteinuria: UACR ≥ 30 mg/g. For ABSI, we divide it by quartile cutoff points. eGFR was estimated from a simplified equation developed from data from the Modification of Diet in Renal Disease (MDRD) study as follows (14): eGFR (ml/min/1.73 m^2^) = 186 × [SCr (mg/dl)/88.4]^-1.154^ × (age)^-0.203^ × (0.742 if women) × 1.233.

### Statistical methods

We performed the Kolmogorov–Smirnov test to explore whether the continuous variables were normally distributed. Continuous variables were presented as mean ± SD or median (IQR) for skewed variables; the classification variables were represented by percentage (%). The Mann–Whitney *U* test was used to compare the difference between continuous variables, and the Chi-square test was used to compare the categorical variables. Logistic regression analysis was conducted to estimate odds ratios (ORs) and 95% confidence intervals (Cis) to determine the association between the ABSI quartile and increased proteinuria, with the lowest quartile as the reference group. We identify conf variables for the relationship between ABSI and albuminuria by reviewing the literature and drawing the DAG. After the univariate analysis between the confounders and UACR, the confounders with a *p*-value less than 0.2 were included in the final model, and the multiple logistic regression model of ABSI and all potential confounders was established, and the optimal model was fitted by stepwise backward regression method. To further investigate the association between ABSI quartile and increased risk of proteinuria, the relationship between gender, age (<60/≥60 years), and eGFR (<90/≥90 ml/min/1.73 m^2^) was stratified. The software used for data analysis was SPSS Version 25.0 (IBM, Chicago, IL, USA). The results were considered statistically significant if the bilateral *p*-value < 0.05.

## Results

### Clinical characteristics of study participants

A total of 40,726 participants were recruited for the study; 29.9% were men, and 70.1% were women. The average age of participants was 58.3 ± 9.26. **Table 1** shows the clinical and biochemical demographics of the subjects, which are divided into two groups based on whether their UACR is elevated or not. Compared with the normal UACR group, the UACR ≥ 30 mg/g group was older, had higher rates of DM and CHD, and had higher LDL-C, WC, GGT, AST, BMI, HbA1c, FBG, PBG, SBP, DBP, and lower HDL-C, education level, and eGFR values (all *p*< 0.001)

**Table 1:** Characteristics of the study population by UACR category.

Variables	UACR < 30 mg/g	UACR ≥ 30 mg/g	*p*-value
*n*	35,208	5,518	
Age, years	57.70 ± 9.02	61.43 ± 10.07	<0.001
Men, %	10,732 (30.5%)	1,436 (26%)	<0.001
BMI, kg/m^2^	24.42 ± 3.70	24.94 ± 3.85	<0.001
WC, cm	85.00 (79.00,92.00)	87.00 (80.00,94.00)	<0.001
SBP, mmHg	127.67 (116.00,141.67)	137.33 (122.00,153.33)	<0.001
DBP, mmHg	76.00 (69.67-83.33)	78.33 (71.00,86.67)	<0.001
TC, mmol/L	5.03 (4.29,5.77)	4.93 (4.20,5.68)	<0.001
TG, mmol/L	1.32 (0.94,1.90)	1.54 (1.08,2.22)	<0.001
HDL, mmol/L	1.30 ± 0.34	1.26 ± 0.33	<0.001
LDL, mmol/L	2.81 (2.23,3.42)	2.92 (2.34,3.54)	<0.001
FBG, mmol/L	5.50 (5.10,6.09)	5.78 (5.20,6.90)	<0.001
PBG, mmol/L	7.30 (6.00,9.41)	8.47 (6.59,12.10)	<0.001
HbA1c, %	6.04 ± 0.93	6.52 ± 1.48	<0.001
AST, U/L	20.00 (17.00,24.00)	21.00 (17.00,26.00)	<0.001
GGT, U/L	20.00 (14.00,30.00)	21.00 (15.00,35.00)	<0.001
eGFR, ml/min	114.89 (102.73,128.79)	109.98 (96.76,125.11)	<0.001
Education level			
Less than high school, %	17,756 (50.8%)	3,173 (57.7%)	<0.001
High school, %	12,714 (36.3%)	1,706 (31.0%)	<0.001
College or more, %	4,512 (12.9%)	620 (11.3%)	<0.001
Smoking habits, %			
No	29,921 (85.0%)	4,804 (87.1%)	<0.001
Occasional	784 (2.2%)	103 (1.9%)	<0.001
Regular	4,185 (11.9%)	575 (10.4%)	<0.001
DM history, %			
Yes	3,229 (9.2%)	1,097 (19.9%)	<0.001
No	31,895 (90.6%)	4,412 (80.0%)	<0.001
CHD history, %			
Yes	1,189 (3.4%)	321 (5.8%)	<0.001
No	33,896 (96.3%)	5,172 (93.7%)	<0.001
ABSI quartiles			
Q1	9,023 (25.6%)	1,060 (19.2%)	<0.001
Q2	8,933 (25.4%)	1,308 (23.7%)	<0.001
Q3	8,877 (25.2%)	1,469 (26.6%)	<0.001
Q4	8,375 (23.8%)	1,681 (30.5%)	<0.001

Data expressed as mean ± SD for continuous variables or median (IQR) for skewed variables and percentage (%) for categorical variables.

BMI, body mass index; WC, waist circumstance; SBP, systolic blood pressure; DBP, diastolic blood pressure; TC, total cholesterol; TG, triglyceride; HDL, high-density lipoprotein cholesterol; LDL, low-density lipoprotein cholesterol; FBG, fasting blood glucose; PBG, 2-h post-load blood glucose; HbA1c, glycosylated hemoglobin; AST, aspartate transferase; GGT, gamma-glutamyl transferase; DM, diabetes mellitus; CHD, coronary heart disease; ABSI, A Body Shape Index; eGFR, estimated glomerular filtration rate.

### Association of ABSI quartiles with increased UACR

**Figure 2:**
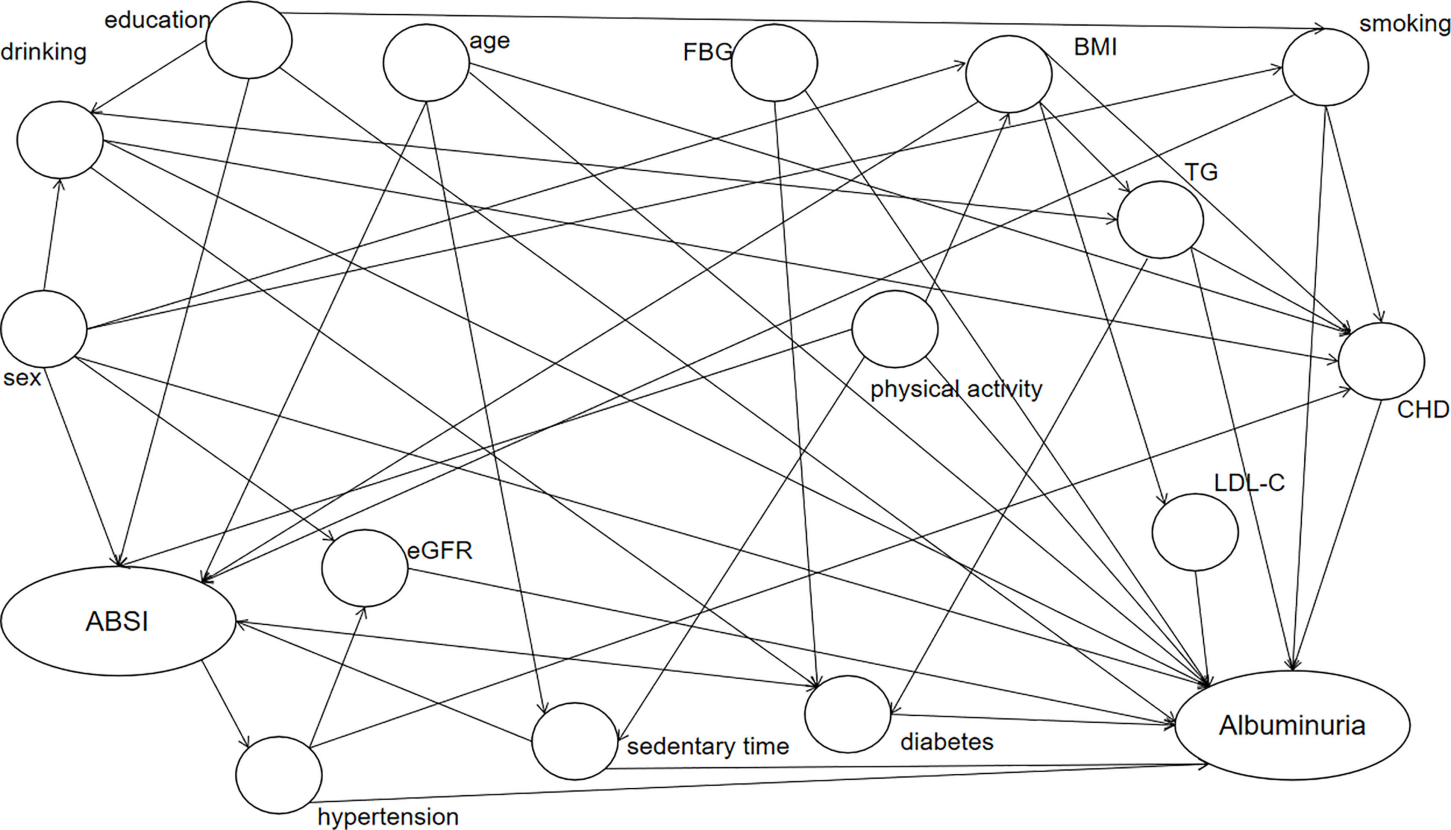
DAG diagram of the ABSI and albuminuria association study. Elliptic nodes represent independent variables ABSI and dependent variables albuminuria, circular nodes indicate possible confounders, and arrows indicate causality. ABSI, A Body Shape Index; BMI, body mass index; TG, triglycerides; LDL-C, low-density lipoprotein cholesterol; CHD, coronary heart disease; eGFR, estimated glomerular filtration rate.

By plotting DAG (**Figure 2**), we found that age, BMI, education level, smoking habits, sex, eGFR, blood pressure level, sedentary time, diabetes, LDL-C, physical activity, TG, FBG, diabetes, CHD, and smoking habits were confounders of the relationship between ABSI and albuminuria. Through single-factor analysis, statistically significant age, sex, smoking habits, CHD history, DM history, education status, blood pressure level, FBG, TG, LDL, and eGFR were selected as confounding factors for multivariate logistic regression analysis to investigate the relationship between ABSI quartiles and UACR elevation (**Table 2**). The correlation of model was significant (OR [95% CI] Q2 vs. Q1: 1.094 [1.001, 1.197]; OR [95% CI] Q3 vs. Q1: 1.126 [1.030, 1.231]; OR [95% CI] Q4 vs. Q1: 1.183 [1.080, 1.295], *p* trend < 0.001).

**Table 2:** Association between ABSI quartiles and UACR in the total population.

Variables	ABSI Quartiles				
	Q1	Q2	Q3	Q4	*p*-value for trend
Model					
OR (95% CI)	1	1.094 (1.001–1.197)	1.126 (1.030–1.231)	1.183 (1.080–1.295)	
*p*-value		<0.049*	<0.009*	<0.001***	<0.001

*p-value < 0.05; ***p-value < 0.001.

Model adjusted for age, sex, smoking habits, CHD history, DM history, education status, blood pressure level, FBG, TG, LDL, and eGFR.

OR, odds ratio; CI, confidential interval; ABSI, A Body Shape Index, CHD, coronary heart disease; DM, diabetes mellitus; FBG, fasting blood glucose; TG, triglycerides; LDL, low-density lipoprotein cholesterol; eGFR, estimated glomerular filtration rate.

### Association of ABSI quartiles with increased UACR in stratified analysis

Stratified analysis was adopted to further verify the stability of the correlation between ABSI and UACR in different populations after comprehensive adjustment of age, sex, smoking habits, CHD history, DM history, education status, blood pressure level, FBG, TG, LDL, and eGFR (**Table 3**). Stratified by sex (*p*-interaction = 0.026), ABSI in the fourth quartile was associated with increased UACR in women (OR [95% CI] Q4 vs. Q1: 1.144 [1.031, 1.268]); however, increased UACR has a significantly association with ABSI in the third and fourth quartile in men(OR [95% CI] Q3 vs. Q1: 1.245 [1.028, 1.507]; OR [95% CI] Q4 vs. Q1: 1.314 [1.080, 1.599]). When subjects have a normal blood pressure (SBP < 120 and DBP < 80) according to Stratification (*p*-interaction < 0.001), the probability of UACR increased gradually from the lowest quartile of ABSI to the highest quartile (OR [95% CI] Q2 vs. Q1: 1.228 [1.038, 1.452]; OR [95% CI] Q3 vs. Q1: 1.200 [1.008, 1.429]; OR [95% CI] Q4 vs. Q1: 1.235 [1.027, 1.485], *p* trend = 0.048). The same trend was observed at the pre-hypertension group (120 ≤ SBP < 140 and/or 80 ≤ DBP < 90) (OR [95% CI] Q3 vs. Q1: 1.229 [1.057, 1.428]; OR [95% CI] Q4 vs. Q1: 1.404 [1.225, 1.610], *p* trend < 0.001). However, the OR was highest in the hypertensive group (SBP ≥ 140 or DBP ≥ 90) (OR [95% CI] Q3 vs. Q1: 1.239 [1.075, 1.429]; OR [95% CI] Q4 vs. Q1: 1.449 [1.249, 1.682], *p* trend < 0.001). Stratified by age (*p*-interaction < 0.001), the elderly (age ≥ 60 years) in Q3 and Q4 were more likely to increase UACR (OR [95% CI] Q3 vs. Q1: 1.183 [1.025, 1.365]; OR [95% CI] Q4 vs. Q1: 1.372 [1.198, 1.572], *p* trend < 0.001). In the younger participants (age < 60 years), ABSI was also significantly associated with increased UACR (OR [95% CI] Q2 vs. Q1: 1.119 [1.001, 1.250]; OR [95% CI] Q3 vs. Q1: 1.142 [1.017, 1.283]; OR [95% CI] Q4 vs. Q1: 1.159 [1.113, 1.185], *p* trend = 0.027).

**Table 3:** Association between ABSI quartiles and UACR in different participants.

Variable	ABSI Quartiles					
	Q1OR (95% CI), *p*-value	Q2OR (95% CI), *p*-value	Q3OR (95% CI), *p*-value	Q4OR (95% CI), *p*-value	*p*-value for trend	*p* for interaction
Gender						0.026
Women	1.0	1.095 (0.991–1.212)	1.121 (1.014–1.206)	1.144 (1.031–1.268)*	0.045	
Men	1.0	1.135 (0.929–1.387)	1.245 (1.028–1.507)*	1.314 (1.080–1.599)**	0.034	
Age, years						<0.001
<60	1.0	1.119 (1.001–1.250)*	1.142 (1.017–1.283)*	1.159 (1.113–1.185)*	0.027	
≥60	1.0	1.104 (0.949–1.285)	1.183 (1.025–1.365)*	1.372 (1.198–1.572)***	<0.001	
BP, mmHg						<0.001
SBP < 120 and DBP < 80	1.0	1.228 (1.038–1.452)*	1.200 (1.008–1.429)*	1.235 (1.027–1.485)*	0.048	
120 ≤ SBP < 140 and/or 80 ≤ DBP < 90	1.0	1.063 (0.912–1.240)	1.229 (1.057–1.428)*	1.404 (1.225–1.610)***	<0.001	
SBP ≥ 140 or DBP ≥ 90	1.0	1.147 (0.991–1.328)	1.239 (1.075–1.429)*	1.449 (1.249–1.682)***	<0.001	

*p-value < 0.05; **p-value < 0.01; ***p-value < 0.001.

Model adjusted for age, sex, smoking habits, CHD history, DM history, education status, blood pressure level, FBG, TG, LDL, and eGFR.

OR, odds ratio; CI, confidential interval; ABSI, A Body Shape Index, CHD, coronary heart disease; DM, diabetes mellitus; AST, aspartate transferase; FBG, fasting blood glucose; GGT, glutamyl transferase; TG, triglycerides; LDL, low-density lipoprotein cholesterol; SBP, systolic blood pressure; DBP, diastolic blood pressure.

## Discussion

In this study, we found that the ABSI levels are associated with increased UACR significantly, and correlation is abated after adjustment of smoking habits, FBG, diabetes history, LDL-C, and CHD history, indicating that history of smoking, blood glucose or lipid metabolic disorders, and history of CHD increase the risk of increased proteinuria in Chinese adults. Furthermore, stratified analysis showed that individuals with higher ABSI levels were more likely to have elevated UACR than those with lower ABSI levels, especially those in the elderly, men, and with hypertension. This study is the first multicenter, large-sample clinical to investigate the relationship between ABSI and UACR in Chinese adults. Early prevention and intervention of proteinuria are crucial; early detection and decreasing abnormal fat distribution may be helpful in preventing adverse outcomes such as CHD, obesity, and DM for patients.

With rapid economic development and lifestyle changes, the incidence of overweight and obesity has increased significantly worldwide. Given this growing trend, it is expected that as many as 57.8% of the population will be overweight or obese by 2030 (15). Obesity is divided into central obesity and peripheral obesity. An increase in visceral fat characterizes central obesity. Excess visceral fat can cause diabetes, hypertension, heart diseases, non-alcoholic fatty liver diseases, kidney disorders, cancer, and other health problems. Traditional anthropometric indicators include WC, BMI, WHtR, and WHR. BMI cannot distinguish fat accumulation from muscle, and WC cannot distinguish visceral fat from subcutaneous fat (16); magnetic resonance imaging (MRI) and computed tomography (CT) are considered to be the gold standard for the distribution of visceral obesity. However, they cannot be routinely used in epidemiological investigations due to the risk of radiation exposure, which is time-consuming and expensive. According to the study (17), ABSI is apparently associated with central obesity and has a better ability to predict type 2 diabetes mellitus (T2DM) than BMI. ABSI has been proved to be associated with all-cause mortality (8), metabolic syndrome (18), DM (19), and hypertension (20). Therefore, ABSI can better measure body size and is expected to become a new standard for health assessment.

The Dutch Prevention of Renal and Vascular End-Stage Disease (PREVEND) study, published in 2003, reported a prevalence of microalbuminuria of 21% or 13%, depending on central or peripheral obesity patterns (21). In a cross-sectional study of adults with T2DM, visceral obesity was significantly associated with UACR (22). Similarly, a follow-up study of 2,393 participants over 4 years observed that participants who had increased visceral fat mass had higher albuminuria (23). Notably, few studies have investigated the relationship between ABSI and proteinuria in individuals who are most likely to develop CKD and have underlying cardiovascular risk factors. Munkhaugen conducted a 20-year cohort study in Norway that assessed 75,000 volunteers and found a strong correlation between BMI and CKD risk, with obese people more likely to develop kidney disease (24). In another large population-based case–control study reported by Ejerblad, patients with a BMI of 25 kg/m^2^ at age 20 had a threefold increased risk of new kidney disease, even after adjusting for hypertension and DM (25). In our study, which included 40,726 Chinese adults, we found that higher visceral obesity as assessed by ABSI was independently associated with an increased risk of proteinuria. These results are consistent with previous studies.

Multiple biological mechanisms may mediate the association between obesity and proteinuria. Recent studies have shown that adipose tissue can secrete adipose tissue-derived adipokines (26) and cytokines (27), such as leptin, which has local effects on mesangial cells, podocytes, and renal tubules, promoting glomerular hyperfiltration (28), which is an independent predictor of proteinuria (29). Participate in the pathogenesis of CKD. In addition, mechanisms such as insulin resistance (30), oxidative stress (31), systemic chronic low-level inflammation (32), and inappropriate activation of the renin–angiotensin–aldosterone system (33) are also involved in developing proteinuria. Prevention of REnal and Vascular ENd-stage Disease study data showed that men have a higher urinary albumin excretion, a known factor for progression of CKD, at any given age, plasma glucose, and BMI than women (34). Our data also showed a gender difference, and we speculated that this might be due to potential anti-fibrotic and anti-apoptotic effects of estrogen or deleterious pro-inflammatory effects of testosterone, as evidenced in animal studies (35). Moreover, age is a well-known independent risk factor for renal impairment, and albuminuria is more likely to occur in the elderly than in middle-aged adults (36). Chronic hypertension can lead to gradual thickening of the glomerular arteries, which can lead to atherosclerotic changes, decreased renal blood flow, decreased kidney function, and decreased filtration of the kidneys, thus leading to increased albuminuria.

Our study provides additional evidence to confirm the association between visceral fat and proteinuria as assessed by ABSI and demonstrates the value of ABSI as a simple, reliable, and effective screening tool for kidney disease risk. In clinical practice, improvements in the distribution and deposition of visceral fat, rather than just weight loss, should be proposed to reduce the associated risk of kidney disease and cardiovascular disease. We believe that ABSI should be used as part of a management strategy to reduce the risk of kidney disease in further clinical practice.

As we know, this study is the first cross-sectional study to explore the relationship between ABSI and elevated UACR with a large sample. However, some limitations need to be considered: First, it cannot clarify the causal relationship between ABSI and elevated UACR as it is a retrospective cross-sectional study, so further prospective studies are necessary. Secondly, the participants were all enrolled from China and were older than 40 years old, so our conclusions may not be applied to other regions and people. Finally, because the application of MRI or CT in such a large population is expensive and inconvenient, our study did not accurately assess visceral adipose tissue. However, previous studies have demonstrated the apparent association between ABSI and visceral adipose tissue, and we suggested that ABSI has the potential to be a reliable and simple tool in proteinuria screening for high-risk people.

## Conclusion

This study demonstrated that increased ABSI levels positively correlated with elevated UACR among adults in the Chinese community. Albuminuria was increased in men, the elderly, and hypertension. Therefore, ABSI can be used as a clinical tool to identify the high-risk population of CKD in Chinese adults. Considering the significant association between visceral fat, proteinuria, and CKD, we should pay more attention to obese individuals and guide them to change their lifestyle and regular exercise, and weight loss is not the only significant benefit of reducing visceral fat deposition.

## Data availability statement

All data used to support the conclusions of the study are not freely available in the view of the privacy principle of Chinese PLA General Hospital, and of protecting the privacy of participants.

## Ethics statement

Written informed consent was obtained from the individual(s) for the publication of any potentially identifiable images or data included in this article.

## Author contributions

YZ analyzed the data and wrote the manuscript. WG, BL, and YL provided great help in the operation and application of SPSS. KC, AW, XT, LY, ZL, GQ, LC, QW, ZG, WW, and GN offered advice and assistance. YM contributed by revising the article. All authors contributed to the article and approved the submitted version.

## Acknowledgments

The authors would like to thank the participants in this study.

## Conflict of interest

The authors declare that the research was conducted in the absence of any commercial or financial relationships that could be construed as a potential conflict of interest.

## Publisher’s note

All claims expressed in this article are solely those of the authors and do not necessarily represent those of their affiliated organizations, or those of the publisher, the editors and the reviewers. Any product that may be evaluated in this article, or claim that may be made by its manufacturer, is not guaranteed or endorsed by the publisher.
